# Art as a generative mediating space: a configurable framework for adolescent affective–sexual learning

**DOI:** 10.3389/fpsyg.2026.1806202

**Published:** 2026-05-15

**Authors:** Xiang Meng, Zichen Ke

**Affiliations:** 1 School of the Art and Design, Anyang Institute of Technology, Anyang, Henan, China; 2 School of the Arts, Universiti Sains Malaysia, Penang, Malaysia

**Keywords:** adolescence, affective–sexual education, arts-based learning, generative learning, mediating space

## Abstract

In response to the structural constraints shaping adolescents' affective–sexual learning across formal and non-formal educational contexts, this paper offers a theoretical reconceptualization of the role of arts-based practices within such learning processes. The paper moves beyond treating art as a tool for expression or information transmission and instead conceptualizes it as a generative artistic mediating space. This space is constituted through the mutual articulation of generativity and spatiality. Building on this conceptualization, the paper proposes a configurable generative conditions framework organized around three relational dimensions: learners' participation positions, the openness of learning issues, and facilitators' relational roles. The framework provides an analytic tool for examining how arts-based practices are configured across educational contexts as generative artistic mediating spaces in which learning processes are not prematurely closed by predefined objectives. It also offers a conceptual perspective for future research and practice that foregrounds the relational conditions and contextual configurations through which generative learning processes unfold.

## 1 Introduction

Adolescence constitutes a critical developmental period during which individuals engage in ongoing exploration and meaning-making related to emotions, sexuality, intimate relationships, and sexual identity. This process unfolds within contemporary social contexts in which young people encounter challenges such as sexual exploitation, abuse, and marginalization, alongside fragmented and often inconsistent sources of affective–sexual knowledge (McKee, 2012; Young et al., 2024). It is a key stage for developing a healthy sense of identity, understanding intimacy, and establishing interpersonal boundaries (Cortínez-López et al., 2021; Habib et al., 2023; Zafra-Agea et al., 2024). In this context, educational processes that support adolescents' affective–sexual development become particularly important. Affective-sexual education is commonly defined as a comprehensive learning process encompassing emotional, relational, bodily, and social dimensions. Its aims extend beyond the transmission of biological knowledge or risk prevention to supporting the development of emotional awareness, relational negotiation, consent literacy, and self-identity as core psychological and social capacities (Goldfarb and Lieberman, 2021; Mullinax et al., 2017; UNESCO., 2024; WHO, 2023). In recent years, research has further expanded theoretical and practical perspectives based on this consensus, gradually shifting attention from biological knowledge and risk prevention toward more holistic dimensions such as affective experience, relational processes, sexual rights, and subjectivity (Cacciatore et al., 2019; Cense, 2019; Goldfarb and Lieberman, 2021; Kågesten and van Reeuwijk, 2021; Todesco et al., 2023).

However, when approaches characterized by cross-system and comprehensive integration are introduced into formal education systems, their implementation at the level of learning organization often encounters significant structural constraints (Fedele et al., 2025; UNESCO., 2024). Under the influence of institutional structures, curricular arrangements, and assessment mechanisms, such approaches may appear reasonable in certain respects, yet they frequently fail to engage with adolescents' complex, fluid, and highly contextualized emotional and relational experiences. In some cases, they may even inhibit deeper learning by eliciting shame, avoidance, or formalized participation, particularly when addressing highly sensitive topics such as gender and sexual diversity or intimate violence (Bellemare-Lepage et al., 2025; Goldfarb and Lieberman, 2021; Pound et al., 2016; Stepień and Mhórdha, 2023).

In contrast to classroom-based and formal modes of learning organization, adolescents' meaning-making around emotions and sexuality often takes place outside the classroom and is distributed across diverse learning contexts, including peer interactions, community spaces, digital media, and participatory projects (Arnett, 2000; Coleman, 2011; Darling and Steinberg, 2017; Gamson and Hertz, 2023; Lameiras-Fernández et al., 2021; Zheng and Han, 2025). These non-formal learning contexts differ from formal classrooms in terms of openness, relational density, and modes of experience sharing, and they often function as important sites of affective–sexual learning during adolescence (Chu et al., 2015; Meehan, 2025).

Accordingly, adolescents' affective–sexual learning is not confined to a single educational setting but unfolds across formal and non-formal environments through the interweaving of multiple contexts, forming a continuous and nested learning ecology. However, substantial differences in content orientation, value frameworks, and modes of support across these learning contexts often make it difficult for adolescents to access sustained and supportive learning conditions that enable effective meaning-making and judgment development (Fedele et al., 2025).

Against this backdrop, arts-based practices have increasingly been incorporated into research and practice related to affective–sexual education, where they are commonly regarded as learning media that support emotional expression, relational reflection, and the provision of psychological safety and symbolic distance in relation to sensitive topics (Bellemare-Lepage et al., 2025; Lys et al., 2018). A growing body of case-based and participatory research has demonstrated the potential of art to support adolescents' exploration of emotions, relationships, and identity-related issues (Bellamy et al., 2025; Burkholder and Keehn, 2025; Renold and Timperley, 2024; Ringrose and Regehr, 2023). Nevertheless, existing studies largely remain at the level of methodological or functional justification, with limited attention to the spatial conditions that art opens up across the learning process as a whole. These insights have also not yet been systematically integrated into a theoretical framework capable of explaining how, and under what conditions, art operates as a core learning structure. This paper shifts the analytical focus away from evaluating the effectiveness of art or prescribing the design of specific artistic activities, and instead focuses on the relational and contextual configurations through which arts-based practices operate as generative mediating spaces that support adolescents' ongoing meaning-making in relation to affective–sexual issues.

On this basis, the paper proposes the concept of the generative artistic mediating space as an integrative theoretical unit and further develops a generative conditions framework centered on relational configuration. The aim of this paper is to reconceptualize how arts-based practices function as generative artistic mediating spaces in adolescents' affective–sexual learning. To this end, the paper adopts a conceptual and interpretive approach to articulate the relational conditions under which such spaces operate across formal and non-formal contexts. The analysis does not take learning outcomes, methodological types, or participation formats as its analytical starting point, but focuses instead on how relational conditions are organized. Through three key relational dimensions, the paper explains how arts-based practices are constituted as mediating spaces across both formal and non-formal contexts, thereby supporting adolescents' ongoing meaning-making in relation to affective–sexual issues.

Within the context of this paper, generativity does not refer to a singular or predefined learning outcome. Rather, it describes affective–sexual learning as a process-oriented practice in which meaning-making, relational judgment, and subjective understanding are not predetermined, but emerge through ongoing practical interactions and are gradually formed under specific relational and contextual conditions (Biesta, 2015; Osberg and Biesta, 2007). The framework provides an analytic tool for examining how arts-based practices are configured as generative mediating spaces across formal and non-formal educational contexts.

## 2 Conceptual and methodological approach

This paper develops a conceptual and interpretive framework examining how arts-based practices function as generative artistic mediating spaces in adolescents' affective–sexual learning. The study is guided by the following research questions: (i) how can arts-based practices be conceptualized as generative artistic mediating spaces, and (ii) under what relational conditions do such spaces support adolescents' affective–sexual learning?

This study adopts a theoretically informed interpretive synthesis approach that integrates insights from three intersecting fields: affective–sexual education, arts-based learning, and sociocultural perspectives on learning. Literature selection is guided by conceptual relevance, with emphasis on studies that explicitly address arts-based practices in affective–sexual or closely related educational contexts and that provide theoretically or interpretively grounded accounts of learning processes. This approach is broadly consistent with traditions of theoretical synthesis and interpretive review in educational research (Torraco, 2005; Snyder, 2019).

The framework was developed through a three-stage process. First, the concept of the generative artistic mediating space was formulated through the integration of the notions of generativity (Biesta, 2015; Osberg and Biesta, 2007) and spatiality in arts-based practices (Ellsworth, 2005; Renold and Timperley, 2024). Building on this conceptual foundation, key relational conditions were interpreted and articulated through engagement with theoretical and empirical studies across formal and non-formal contexts. These conditions were then synthesized into a configurable framework organized around three core dimensions: learners' participation positions, the openness of learning issues, and facilitators' relational roles.

The framework is situated in relation to established traditions in educational theory, including sociocultural perspectives on learning, critical pedagogy, and generative approaches to education, while extending these perspectives by foregrounding the relational configuration of generativity and spatiality in arts-based affective–sexual learning contexts. In addition, this study remains attentive to a growing body of work from the Global South that highlights how arts-based approaches are used to engage with culturally sensitive issues such as sexuality, gender, and social violence in contextually grounded ways (Elliott, 2024; Hoosain Khan, 2014; Khan and Marnell, 2022).

## 3 Conceptual grounding: generative artistic mediating space

### 3.1 Reframing affective-sexual learning as a generative process

In existing affective-sexual education practices, complex issues related to emotion, sexuality, and relationships are often translated into operationalizable instructional objects in order to be organized and implemented within formal education systems (Fedele et al., 2025). Shaped by institutional structures, curriculum arrangements, and assessment mechanisms, a results-oriented logic of learning organization tends to exert a strong influence at the level of curriculum implementation and assessment. Within this logic, learning is understood as the guidance or correction of knowledge, attitudes, or behaviors through standardized content, predefined objectives, and measurable learning indicators (Bellemare-Lepage et al., 2025; UNESCO., 2018). This approach often manifests as a prioritization of identifiable and measurable risks or problem domains, such as health and risk prevention, with teaching centered on mental health interventions, the prevention of risk behaviors, and reproductive health issues including sexually transmitted infections and unintended pregnancy (Fedele et al., 2025; Zafra-Agea et al., 2024).

However, adolescents often find it difficult to connect such curricular content to their own lived experiences. They commonly report that affective-sexual education simplifies, avoids, or remains silent on key emotional and relational experiences, thereby failing to adequately support their understanding of desire, identity formation, emotion, and intimate relationships as complex issues (Alekseeva et al., 2015; Burns, 2023; Molina et al., 2015; Pound et al., 2016). At the same time, the lack of systematic teacher training and the absence of clear institutional guidelines have been repeatedly identified as factors that further constrain the depth and quality of classroom engagement with these topics (Plaza-del-Pino et al., 2021; Rubio Fernández et al., 2025).

Within this context, adolescents' affective-sexual learning has not been fully integrated into formal education. It occurs extensively within non-formal learning spaces such as peer interactions, online communities, and digital media environments (McKee, 2012; Stein et al., 2022; Young et al., 2024). Although these contexts play a significant role in information access and experience sharing, the quality and value orientations of their content are neither consistently positive nor stable. Some information is contradictory or misleading, including exposure to pornographic content (Litsou et al., 2021). As a result, across both formal and non-formal educational contexts, affective-sexual learning is often reduced to transmissible information or singular behavioral templates, thereby weakening its capacity to respond to adolescents' lived and context-specific experiences.

Moreover, sexuality, emotion, intimacy, and identity are not stable learning objects that can be directly transmitted through instructional media. Rather, they constitute processual experiences that are highly dependent on specific relationships, contexts, and learning spaces (Cortínez-López et al., 2021). From this perspective, within generative learning processes, meaning is not transmitted or acquired. Instead, it is gradually generated through concrete interactions and practices, and continuously formed within relational engagement. As noted by Renold and Timperley (2024) in their research on curriculum reform in relationships and sexuality education, learning is not organized around predefined objectives but instead develops through arts-based and creative practices, within which judgments about relationships, meaning, and value orientations emerge through continually evolving experience.

It is important to emphasize that the generativity discussed in this paper does not refer to a self-sustaining or naturally expanding learning state. Instead, it denotes a highly context-dependent process whose emergence is always embedded within specific institutional arrangements, relational structures, and emotional conditions. Generativity is not defined by stability or replicability, but takes different forms in practice as institutional pressures, relational dynamics, and emotional conditions shift.

### 3.2 Art as a dual mediating space of generativity and spatiality in affective-sexual learning

Affective-sexual learning, understood as a generative learning process, centers on the gradual formation of understandings of sexuality, identity, and related issues through the ongoing reworking of relationships and practices within specific contexts (Biesta, 2015; Osberg and Biesta, 2007; Wenger-Trayner and Wenger-Trayner, 2014). Although arts-based practices have been widely introduced into affective-sexual education and have demonstrated substantial potential, this paper does not focus on specific methods or functional effectiveness. Nor does it limit art to a means of facilitating individual expression or transmitting information. It examines how art constitutes generativity and spatiality as the conditions that enable this learning process to unfold.

In this paper, “space” does not refer merely to a physical setting, but to a mediating field formed through psychological safety, social relations, and expressive practices in art. This understanding of space resonates with broader theoretical discussions of relational and socially produced space within educational and cultural theory (Ellsworth, 2005), while extending them by emphasizing how generativity and spatiality are co-constituted within arts-based affective–sexual learning processes. Similarly, “art” does not refer to a specific genre or medium, but to a mode of artistic practice characterized by open forms of expression. Although different art forms may introduce subtle differences in how such a mediating space is constructed, the analytic focus of this paper is not on comparing forms, but on the relational conditions that underpin their operation. Based on these definitions, art functions as a mediator in affective–sexual learning precisely because it serves a dual role throughout the learning process, namely generativity and spatiality. The following discussion develops these two interrelated analytic dimensions to clarify how art supports affective-sexual learning as a generative process.

First, from the perspective of generativity, art offers adolescents a non-linear learning pathway without prespecified outcomes. It enables understandings of emotions, relationships, gender, and sexual orientation to emerge gradually through ongoing processes of expression, response, and practice. Existing studies suggest that generative artistic processes help adolescents recognize and regulate emotional experiences related to intimacy, gender, and identity. They also promote reflection on self and relational understanding, and support the development of more complex self-concepts, communication capacities, and self-efficacy within peer interactions (Deer et al., 2023; Kastner et al., 2021; Lee and Lee, 2021). This process is not oriented toward arriving at correct answers. Rather, it supports learners in relating their own experience to others' perspectives and to multiple social narratives, thereby gradually generating understandings of sexuality and relationships, as well as developing judgments and self-understanding related to identity (Huuki et al., 2022; Renold et al., 2026).

Second, from a spatial perspective, art provides a necessary contextual container for generative processes by creating a mediating space characterized by psychological safety and a buffering function, allowing learners to pause, explore, and reflect without being required to immediately articulate, justify, or assume personal positions in relation to sensitive issues (Dickson, 2021; Rouse et al., 2023). As Renold and Timperley (2024) have noted, arts-based practices provide adolescents with a space of temporary shelter in which they can dwell with issues related to intimacy, gender, and identity. This space is characterized by permeability and cross-contextuality, enabling it to traverse the boundaries of classrooms, community settings, and digital environments, and allowing learning to continue across multiple social ecologies (Puutio et al., 2023; Renold and Timperley, 2023).

It is important to emphasize that generativity and spatiality are not independent properties. They are mutually constitutive and mutually enabling. The new modes of understanding and relational perspectives opened through artistic practice can unfold only within a mediating space that allows for dwelling, delay, and continuous adjustment. Conversely, the educational significance of such a space lies precisely in its capacity to sustain and propel generative exploration, not in directing learners toward closed or predetermined answers. In this sense, art can be understood as a mediating space constituted through the joint operation of generativity and spatiality, providing an analytic lens for examining how affective-sexual learning is supported across different learning contexts.

Furthermore, the educational potential of art as a generative artistic mediating space does not derive from static elements or fixed operational pathways, such as predefined activity formats, standardized instructional procedures, or uniform learning objectives. Instead, it depends on the dynamic configuration of multiple relational conditions within specific contexts, including learners' positions of participation, the openness of learning issues, the relational positioning of facilitators within the learning space, and the conditions of artistic practice and expression. These conditions are understood as analytic perspectives for examining how generative artistic mediating spaces emerge and operate across different educational contexts.

**Figure 1:**
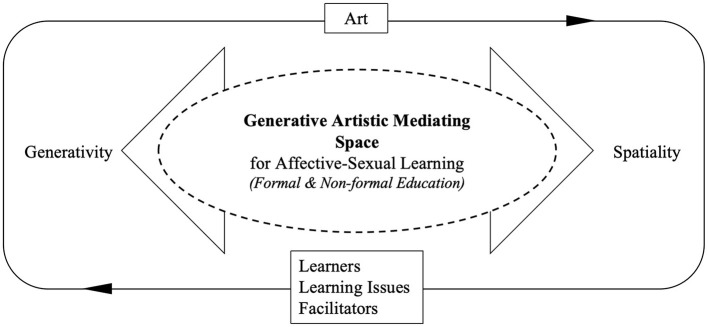
Art conceptualized as a mediating space constituted through the reciprocal interaction between generativity and spatiality. The outer loop indicates the ongoing and cyclical nature of arts-based learning processes across contexts. The three elements at the base represent key relational conditions: learners, learning issues, and facilitators.

As illustrated in Figure 1, art is conceptualized as a mediating space constituted through the interaction of generativity and spatiality. The bidirectional arrows indicate their reciprocal relationship, while the central area represents the space within which affective–sexual learning unfolds across formal and non-formal contexts. This space does not emerge from predefined objectives or prescribed operational pathways, but comes into being through the configuration of relational conditions within specific contexts. The following section will further examine the key relational conditions that support the generation of this mediating space.

### 3.3 Relational conditions of the generative artistic mediating space: the configuration of learners, issues, and facilitators

Within the context of affective-sexual education discussed in this paper, learners primarily refer to adolescents. However, the analysis in this section is not confined to a specific age group. Rather, it focuses on learners' participation positions and relational configurations within the generative artistic mediating space. A generative artistic mediating space is generated through the configuration of multiple relational conditions within specific contexts. From this perspective, the analysis focuses on three relational conditions that recur across different learning settings and exert a decisive influence on generativity. These conditions are learners' participation positions, the openness of learning issues, and facilitators' relational roles. The following subsections examine each of these conditions in turn, analyzing how different configurations jointly shape the extent to which generative artistic mediating spaces can support affective–sexual learning.

It should be noted that the three conditions identified here are not proposed as exhaustive categories. Rather, they are derived through conceptual integration of key analytic orientations found in sociocultural learning theory, research on generative learning, and critical pedagogy (Biesta, 2015; Ellsworth, 2005; Freire, 2020; Lave and Wenger, 1991). The purpose of this framework is not to establish a universally applicable normative structure. Instead, it seeks to illuminate how art may be transformed into a mediating space that supports the ongoing emergence of meaning when this constellation of relational conditions is reconfigured in practice and oriented toward generative principles.

#### 3.3.1 Learners: from passive recipients to generative participants

Learners' participation positions within the learning space constitute a key relational condition that shapes whether generative learning can unfold. When adolescents are presupposed as lacking knowledge or as inherently vulnerable to risk, they are positioned as carriers of problems whose role is limited to internalizing and receiving predefined information and norms. Within affective-sexual education and related psychoeducational discourses, learners are thus repeatedly framed as objects to be shaped and managed (Allen, 2005; Fine and McClelland, 2006; Romer, 2003). Under this configuration, the learning space is implicitly constructed as a closed channel of transmission, and artistic practices are likely to be reduced to technical activities that illustrate predetermined conclusions. This constrains the possibility for learners' own experiences and questions to function as starting points for learning.

By contrast, the generative artistic mediating space emphasizes a reconfiguration of learners' participation positions. Learners are understood as active participants through whom learning unfolds, rather than as objects in need of correction. This understanding is consistent with broader educational discussions on learner agency (Freire, 2020), as well as with sociocultural learning theories that examine the relationship between participation positions and learning as a generative process (Biesta, 2015; Lave and Wenger, 1991). From this perspective, learners' personal experiences, bodily sensations, and complex emotions are no longer treated as content to be avoided or corrected. Instead, they become key resources that propel the learning process forward.

In this sense, the distinctive contribution of art lies in providing an indispensable practical pathway for this shift in learner positioning. Through its non-verbal and polysemic qualities, art offers symbolic forms through which inner experiences that are difficult to articulate or not yet fully formed can be externalized and rendered perceptible (Brandt, 2006; Fedele et al., 2025). This is particularly significant in affective-sexual learning, where adolescents often require a degree of psychological distance in order to participate without being exposed to shame, judgment, or categorization pressures (Ellsworth, 2005). For example, in artistic practices such as drawing, collage, or drama, personal emotions, experiences, and narratives can first be held within the artwork itself (Visser and Du Plessis, 2015). This reduces the pressure associated with expressions that directly point to personal positions or lived experiences, and creates a safe mediating object for subsequent reflection and deepened understanding (Bosgraaf et al., 2020). In this process, learners are not reproducing existing answers. Rather, they engage in exploratory forms of generative expression through their artworks, gradually forming understandings of the relevant issues at their own pace along perceptual and reflective pathways opened by artistic practice (Renold and Timperley, 2024).

Accordingly, the practical organization of the learning space often reflects a redefinition of how learners participate. Art provides learners with a visualized mode of participation that does not require direct verbal articulation of personal experience or fixed positions (Eisner, 2003; Feinstein, 1985). This enables learners, while maintaining psychological safety, to externalize emotions, sexuality, confusion, and relational experiences as objects that can be explored and discussed collectively. For instance, learners may be invited to use clay or bodily-based artistic forms to express experiences and boundaries related to closeness and withdrawal in intimate interactions, or to explore situations involving sexual harassment (Bosgraaf et al., 2020; Hetherington and Gentile, 2022; Sholt and Gavron, 2006; Weinrabe et al., 2025). The core function of such practices lies in enabling learners to transform inner experiences into shared objects of inquiry that can be observed, reflected upon, and jointly explored at a safe distance.

In sum, within a generative artistic mediating space, the key transformation of learners lies not in expression or content reception *per se*, but in whether they are positioned to participate in a generative learning process rather than to complete predefined tasks. When learners move from passive recipients of knowledge to generative participants who engage in symbolic exploration and expression through art, a generative artistic mediating space begins to emerge, continuously giving rise to unpredictable generative potential through processes of creation and presentation. This transformation constitutes the primary relational condition through which the generative artistic mediating space comes into being, and it provides a key point of reference for assessing whether generativity has been activated in practice.

#### 3.3.2 Learning issues: from predetermined problems to open issues

An open issue does not refer to a discussion without direction. Rather, it denotes an issue whose pathways of understanding and outcomes of judgment are not closed in advance, but are gradually developed through the learning process. Core topics in affective-sexual learning, such as desire, consent, power, and identity, are shaped to a large extent by how they are presented, which in turn determines the depth of exploration that learning can reach (Biesta, 2015; Ellsworth, 2005). Within conventional instructional models, these topics are often framed as closed problems to be taught, accompanied by predefined normative positions or correct answers (Goldfarb and Lieberman, 2021; Pound et al., 2016). When addressing complex issues related to gender and sexuality education, this problem-oriented configuration tends to reduce learning to assessable cognitive objectives. As a result, discussions are directed toward consensus, while difference, hesitation, and uncertainty are frequently treated as elements to be corrected or eliminated. From the perspective of generative conditions, learning issues themselves therefore constitute a key condition that must be continuously opened, examined, and adjusted in practice.

A generative artistic mediating space requires learning topics to be reconfigured as open issues, not as problems with predetermined conclusions. Within this framework, the core role of art is not to illustrate or explicate normative propositions, but to transform abstract and highly sensitive affective- and sexuality-related issues into exploratory materials that begin from sensory experience, hands-on engagement, or collective discussion (Berleant, 2011). Through artistic creation and participation, learners enter concrete and lived processes of meaning formation, gradually developing contextual judgment about which relational configurations can be sustained and which forms of interaction require adjustment (Renold and Timperley, 2024). Art extends across the duration of a project or course, enabling learners to continuously form and revise judgments rather than simply comply with established rules.

In this process, learning topics no longer appear as propositional knowledge to be memorized or mastered. Instead, they are constituted as open issues that can be experienced, shaped, and discussed as learning materials. They are not introduced through prescriptive definitions of what should or should not be done. Rather, they are rendered perceptible and intelligible through bodily sensations, emotional responses, and relational interactions within artistic practice. Art provides an anchor for the unfolding of thinking and feeling, enabling learners to develop understandings of these issues in a slow, non-linear manner that remains closely connected to their own experiences (Dickson, 2021; Eisner, 2003; Rouse et al., 2023).

In sum, within a generative artistic mediating space, the central concern regarding learning issues does not lie in whether they are taught clearly. Instead, it lies in whether they retain sufficient openness and investigability to continuously attract and sustain the engagement of diverse experiences. The educational significance of such issues does not stem from their being definitively defined or normatively resolved at a single moment. Rather, it emerges from their ongoing activation within the learning process, enabling learners to navigate uncertainty, contradiction, and risk in lived contexts, and to gradually develop forms of understanding that remain open to reflection and ongoing adjustment.

#### 3.3.3 Facilitators: as guardians and co-participants of the space

In this paper, the term facilitators refers to adult actors, such as teachers, social workers, and artists, who are able to provide support for adolescents' affective–sexual learning across different contexts. They are not defined by fixed professional identities, but by their participation in specific learning situations, where they engage in relational practices that contribute to maintaining the safety and openness of the generative artistic mediating space. It is important to emphasize that the facilitators discussed here do not include any unprepared adults or those lacking professional and ethical awareness. Rather, they are participants who, within particular educational or practice-based contexts, possess basic capacities for professional judgment, ethical sensitivity, and reflexivity, and who are able to assume supportive roles within institutional and relational boundaries.

In this sense, the role of the facilitator is not grounded in occupational status, but in the mode of support and relational positioning enacted in practice, which is continuously shaped and adjusted through ongoing relational engagement. On this basis, the generative artistic mediating space requires facilitators to move beyond the role of teaching or transmitting normative knowledge, shifting from “guiding toward conclusions” to “holding the space.” As a relational role, the facilitator's function lies in participating in and modulating the conditions through which the space comes into being, rather than directing learners toward predefined understandings or value positions. Instead, the core task is to safeguard the necessary uncertainty, openness, and relational tension within the learning process, so that generative learning can continue to unfold (Biesta, 2015; Ellsworth, 2005).

As guardians of the space, facilitators carry a key responsibility to temporarily suspend normative judgment and immediate response. By loosening authoritative positioning and maintaining a mode of attentive and respectful presence, they create a relational field in which learners are able to dwell, test possibilities, and return to issues repeatedly (Bouton et al., 2024). This temporary suspension of normative judgment does not imply the abandonment of ethical responsibility or the neglect of risk. On the contrary, it operates within clear ethical boundaries and requires facilitators to demonstrate heightened sensitivity and accountability with regard to emotional safety, power asymmetries, and potential harm. Within these parameters, such guardianship is not a passive absence of action, but a highly situated form of relational work. Facilitators regulate interactional tempo, sustain open dialogic structures, accommodate polysemic forms of expression, and establish boundaries when necessary to prevent harm. Through these practices, the learning space is prevented from collapsing into disorder while also avoiding premature closure (Alrø and Billund, 2021; Ruiz-Mesa and Hunter, 2019). Throughout this process, facilitators remain attentive to whether the space continues to allow the coexistence of different experiences and understandings, and they support meaning negotiation by cultivating an atmosphere that is coordinated, relaxed, and nonjudgmental.

At the same time, facilitators also participate in the generative process as co-participants. This means that adults do not manage the learning process from outside, but enter into the exploratory terrain opened by artistic practice together with learners through a mode of presence that is engaged without being directive (Chapin et al., 2022). Their participation is expressed primarily through response, questioning, and shared presence rather than through evaluation or summary. For example, facilitators may offer open-ended responses to artworks, pose extending questions in response to learners' narratives, or gently mark elements that have not yet been articulated. Through such practices, facilitators help sustain the flow of meaning negotiation rather than replacing learners in the task of meaning construction.

In this sense, the educational significance of the facilitator does not lie primarily in what is said, but in what is deliberately not introduced too early. Avoiding the rapid translation of complex or singular experiences into normative language, refraining from moralizing emotional responses, and resisting the compression of polysemy into singular interpretations constitute essential conditions for maintaining the generative artistic mediating space (Bouton et al., 2024). This capacity to hold uncertainty allows learners to continue exploring their feelings and relational understandings from positions that are not yet fully defined, without pressure to arrive at socially sanctioned answers.

Accordingly, within a generative artistic mediating space, the key role of the facilitator is not to shape specific learning outcomes. Rather, it lies in continuously regulating the dynamic balance between safety and generativity through a guarded form of presence and a restrained mode of collaboration. When facilitators successfully enact this role, a generative artistic mediating space is sustained and further constituted, moving beyond a merely physical or conceptual arrangement. It thereby becomes a relational space with psychological salience and generative potential, enabling affective–sexual learning to unfold as an ongoing generative process within relationships characterized by trust and openness. This configuration of the facilitator role constitutes a central enabling condition for the existence of the generative artistic mediating space and serves as a key operational point for maintaining the balance between generativity and psychological safety in practice.

Taken together, the foregoing analyses of learners, learning issues, and facilitators demonstrate that a generative artistic mediating space is not determined by any single element or fixed method. Rather, it is generated through the dynamic configuration of three relational conditions within specific learning contexts. The particular combinations of these conditions form the foundation for the subsequent analysis of the generative conditions framework.

## 4 A configurable generative conditions framework for arts-based affective-sexual education

Building on the preceding analysis of the generative artistic mediating space and its key relational conditions, this paper further proposes a configurable generative conditions framework for understanding how arts-based affective-sexual education practices function as generative artistic mediating spaces across different contexts. The framework shifts analytic attention away from predefined learning outcomes and toward how learning spaces are organized to support meaning-making, relational judgment, and psychological safety. Given that adolescents' affective-sexual learning unfolds across both formal educational settings and non-formal learning contexts, the framework emphasizes the contextual sensitivity and adaptability of condition configurations, allowing the same core dimensions to be mobilized in different ways across diverse educational settings. The following sections outline the framework's core dimensions and scope of application.

### 4.1 Core dimensions of the generative conditions framework

The generative conditions framework supports the analysis and evaluation of how art is configured as a generative artistic mediating space across different learning contexts. Within the context of this paper, “generative conditions” do not refer to specific instructional elements, activity formats, or operational inputs. They refer to relationally configured conditions within specific learning situations through which generativity may emerge, be sustained, or become constrained. Within this framework, the analytical focus does not lie in the formal characteristics of artistic activities or in the design of expressive tasks themselves, but in how relational structures within the learning space are organized.

On this basis, the paper identifies three core analytic dimensions that can be used in practice to assess whether generativity is activated, sustained, or weakened: (1) learners' participation positions, (2) the openness of learning issues, and (3) facilitators' relational roles. These three dimensions do not operate independently. Instead, they interact within specific contexts to jointly shape how the learning space functions and what generative potential it affords. To make these dimensions more analytically tangible, brief illustrative examples are provided below.

First, learners' participation positions constitute the primary analytic entry point for determining whether generativity is initiated. When learners are configured solely as recipients tasked with executing predefined expressive assignments or reproducing prescribed content, their participation remains at the level of task completion, and artistic activities are unlikely to give rise to new meaning. By contrast, when learners are permitted to introduce their own experiences through artistic practice, raise questions that have not been predetermined, and influence the direction of interaction, their participation position can shift toward that of meaning-making participants. Under such configurations, personal experience and emotional perception are able to enter collective exploration, and generativity is thereby activated.

For example, in a classroom-based activity, learners might be invited to draw or stage scenes depicting a moment of uncertainty, such as a character being asked by a peer to do something they are unsure about, without being required to resolve the situation or arrive at a “correct” answer. The artwork becomes a shared reference point for discussion, allowing learners to explore questions of consent, boundary-setting, and peer pressure indirectly through the situation they have created rather than through personal disclosure.

Second, the openness of learning issues constitutes a critical condition for determining whether generativity can be sustained. In affective-sexual learning, when issues are presented as closed problems or as normative conclusions, learning processes tend to converge rapidly toward uniform answers, and generativity correspondingly diminishes. Conversely, when issues are configured as open, negotiable, and reconfigurable, their boundaries of meaning remain unfinished in practice. This enables learners to develop judgment through exploration rather than merely acquiring rules. Whether issues allow multiple interpretations to coexist thus becomes a key indicator for assessing the maintenance of generativity.

For instance, in a community-based arts workshop on affective–sexual issues such as boundaries, consent, and intimacy, participants might be invited to create short performances or visual collages that depict ambiguous situations, such as a character receiving mixed signals in an intimate interaction or hesitating in response to subtle forms of pressure. These artifacts can then serve as shared points of reference for discussion, allowing multiple interpretations to emerge and remain in tension rather than being resolved into a single normative conclusion.

Third, facilitators' relational roles function as a central pivot for determining whether generative conditions can be continuously sustained. When adults intervene prematurely to guide conclusions, clarify positions, or impose evaluative judgments, the learning space is quickly normalized and generative processes are interrupted. In contrast, when facilitators regulate interactional tempo, acknowledge polysemic expression, and deliberately delay normative judgment, their mode of presence preserves time and psychological safety within the learning space. This allows learners to continue exploring experience, revising understanding, and developing judgment from positions that are not yet fixed.

For example, participants may share drawings, short videos, or anonymous visual posts exploring themes such as identity, desire, or boundaries. Rather than immediately interpreting or evaluating these contributions, facilitators may respond by posing open-ended questions, drawing attention to contrasting perspectives, or allowing moments of silence when responses are not readily formed. Such responses create space for ambiguity to persist, enabling learners to remain with uncertainty and continue the process of meaning-making without premature closure.

Taken together, learners' participation positions, the openness of learning issues, and facilitators' relational roles form a dynamic analytic triangle for examining how a generative artistic mediating space is configured in practice. Within this structure, changes in the configuration of any single dimension affect the possibilities of the others. Generativity is not triggered by a single element but emerges through the coordinated operation of multiple relational conditions. For this reason, the generative conditions framework does not offer a replicable best practice model. Rather, it functions as an analytic and judgment-oriented tool for comparing, assessing, and reflecting on how arts-based practices support or constrain adolescents' psychosocial meaning-making processes under specific relational configurations across different learning contexts.

**Figure 2:**
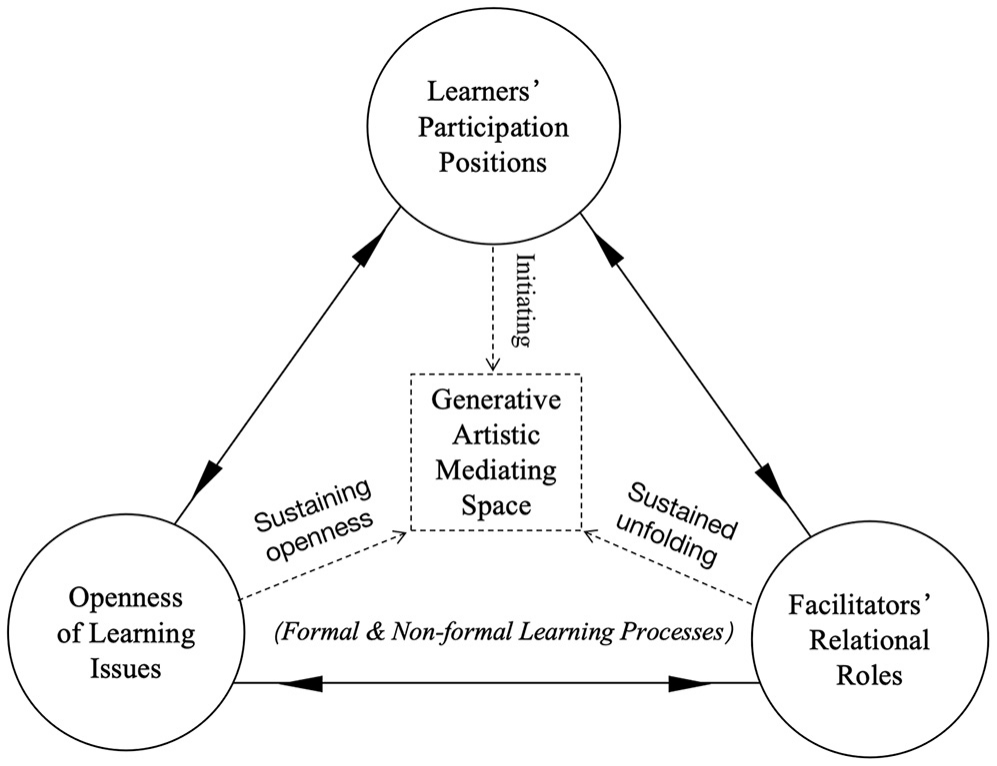
A schematic representation of the generative conditions framework. The triangular structure depicts the interdependence among three core relational conditions: learners' participation positions, the openness of learning issues, and facilitators' relational roles. Arrows indicate their dynamic interaction across formal and non-formal learning contexts.

As illustrated in Figure 2, the generative conditions framework shows how the ongoing interaction among three core conditions, namely learners' positions of participation, the openness of learning issues, and facilitators' relational roles, jointly constitutes a generative artistic mediating space that takes form across both formal and non-formal learning contexts. The triangular structure represents their interdependence, while the arrows indicate their dynamic interaction.

It is important to further emphasize that artistic practice does not automatically carry generative significance by virtue of its formal characteristics alone. When artistic activities become highly task-oriented, outcome-driven, or evaluation-focused, their operational logic may quickly revert to an intervention-oriented mode, transforming the creative process into an instrumental means for achieving predetermined expressive goals or psychological effects. Under such conditions, art no longer functions as a generative artistic mediating space but is instead embedded as a technical medium within existing instructional logics. For this reason, the generative conditions framework does not attribute generativity to the formal properties of artistic media themselves. Rather, it directs analytic attention to how learners' participation positions, the openness of learning issues, and facilitators' relational roles are synchronously configured when artistic practices are introduced.

### 4.2 Configuring generative conditions across formal and non-formal learning contexts

Although formal and non-formal learning contexts differ substantially in their institutional logics, modes of participation, and relational structures, the generative conditions framework does not distinguish between models on the basis of context type. It emphasizes that the emergence of generative artistic mediating spaces depends on a set of core conditions that are transferable across contexts. These conditions require continuous adjustment in response to different institutional and relational constraints.

For example, in some contexts, including parts of Asia, affective–sexual topics may be subject to stronger curricular regulation, moral expectations, or religious sensitivities, which can limit how openly certain issues can be introduced or discussed. Under such conditions, generative spaces may need to be organized more cautiously, with greater attention to indirect forms of expression, symbolic mediation, or gradual opening of sensitive issues. In contrast, in contexts where discussions of sexuality are more institutionally supported and publicly legitimized, issues may be introduced more explicitly, allowing for more direct forms of participation and facilitation to emerge. These variations do not alter the core dimensions of the framework, but shape how they can be enacted and balanced in practice. Generativity is therefore not an inherent property of any particular learning setting, but a processual feature that emerges through how relational conditions are calibrated and organized within specific contexts.

### 4.3 Scope and boundaries of the generative conditions framework

The generative conditions framework is an interpretive, analysis-oriented theoretical tool that takes the organization of conditions as its point of entry. Its purpose is to examine how arts-based practices are configured as generative artistic mediating spaces under specific institutional and relational conditions, and how these conditions shape whether generative learning is sustained, expanded, or constrained.

From a methodological perspective, the framework is intended to make sense of a generative process that is highly dependent on context, relational structures, and situated judgment, rather than as a directly applicable model for practice. Its analytic value lies in supporting researchers and facilitators in attending to how generativity is being maintained, weakened, or prematurely closed during practice, and in understanding how adolescents' psychological formation and meaning-making processes shift in relation to changing conditions.

In terms of applicability, the framework is not confined to affective–sexual learning, but may also be relevant to other arts-based learning contexts where meaning-making processes are open-ended, relationally mediated, and not predetermined by fixed outcomes. It supports comparative analysis of how generative learning spaces are configured across different institutional and relational settings, and under what conditions generativity is sustained or constrained. It should be noted, however, that in family contexts, where relationships are often characterized by strong emotional attachment, power asymmetries, and overlapping boundaries (Bronfenbrenner, 1979; Goldfarb and Lieberman, 2021), the relational conditions central to this framework, including participation positioning, the openness of learning issues, and the facilitator's role, are more difficult to deliberately configure. For this reason, the family is not treated here as a primary analytic or application context for the framework.

Finally, the generative conditions framework is not intended to replace existing curricular structures, professional training systems, or institutional regulations in the field of affective–sexual education. It offers a process-oriented conceptual language that operates within existing institutional frameworks, enabling a more nuanced understanding of how generative conditions are configured in practice.

## 5 Discussion

### 5.1 Theoretical contributions of the generative conditions framework

The generative conditions framework proposed in this paper addresses a key theoretical question in affective–sexual education: how, and under what relational and contextual arrangements, arts-based practices move beyond an instrumental role to constitute a mediating space that supports adolescents' ongoing and emergent affective–sexual learning. Unlike existing studies that emphasize participation, expressive freedom, or pedagogical strategies, this framework shifts attention to the relational conditions under which learning spaces accommodate uncertainty, so that learning processes are not prematurely closed by predefined objectives.

On this basis, the contribution of the framework can be specified across three interrelated and progressively deepening levels. The framework builds on insights from sociocultural learning theory, critical pedagogy, and generative approaches to education, while extending these perspectives by specifying how relational conditions configure generative artistic mediating spaces in practice.

First, in terms of how affective-sexual learning is understood, the framework shifts analytic attention from outcome-oriented conceptions of learning toward a generative process that unfolds continuously within concrete relational and situational contexts. Although existing scholarship has increasingly emphasized relationships, rights, and subjectivity, learning is still often implicitly treated in practice and evaluation as the acquisition of predefined knowledge, attitudes, or behavioral norms (Goldfarb and Lieberman, 2021). The starting point of this framework is the suspension of this assumption, redefining affective-sexual learning as an ongoing process in which meaning and judgment emerge through interaction and relational practice instead of being treated as predefined or fixed outcomes (Biesta, 2015). This shift is crucial because it relocates the core concern of education from how to design learning in order to achieve predefined goals to how to identify and shape relational conditions that allow uncertainty to persist and support the negotiation of meaning. This reconceptualization provides a necessary theoretical foundation for re-anchoring the role of art within affective-sexual education.

Second, at the level of conceptualization, the framework introduces the integrative theoretical unit of the generative artistic mediating space, systematically linking the processual generativity of art with its relational spatiality. Existing studies often treat art as a functional medium for facilitating expression, enabling safe discussion, or transmitting information (Bellemare-Lepage et al., 2025). The framework instead suggests that the core educational significance of art lies in the distinctive relational field it constitutes. Within this field, the generativity of art, understood as supporting nonprescriptive exploration and the emergence of meaning, and its spatiality, understood as providing symbolic distance and a container for psychological safety, are mutually constitutive and mutually reinforcing. Generativity requires space to hold uncertainty, while the educational vitality of that space derives precisely from its ongoing capacity to host and catalyze generative exploration. Through this conceptualization, art is elevated from an auxiliary method to a central structural component within the learning ecology.

Third, at the level of analysis and interpretation, the framework develops a condition-based configuration model with a high degree of contextual sensitivity for understanding how generative learning spaces are constructed, sustained, or constrained in practice. The framework does not present static or idealized blueprints for practice. Instead, it distills three core relational dimensions that recur across contexts and are mutually constitutive: learners' participation positions, the openness of learning issues, and the relational roles of facilitators. This structure enables researchers and practitioners to move beyond surface-level judgments of activity form or participatory atmosphere and instead examine whether learners' experiences are treated as starting points for meaning generation, whether learning issues remain under exploration or are prematurely closed, and whether facilitators' interventions sustain the openness of the space or redirect it toward normative conclusions. At the same time, the framework explicitly acknowledges that generativity is not an automatic outcome of artistic practice, but the product of ongoing negotiation among relational conditions within specific institutional, cultural, and power structures. For example, in formal educational settings, time constraints and accountability pressures often compress the conditions required for issues to remain open. On this basis, the framework does not seek a uniform degree of openness or a fixed depth of facilitation. Instead, it supports a situated mode of judgment in which key decisions are dynamically adjusted in response to how relational conditions interact in the immediate context, thereby maintaining a necessary balance between generative exploration and psychological safety.

Taken together, the core theoretical contribution of the generative conditions framework lies in offering a coherent set of conceptual tools oriented toward explaining the complexity of practice. Instead of providing simple prescriptions for ensuring success, the framework offers an analytic lens for understanding how success becomes possible and how difficulties emerge. In doing so, it provides a foundation for future research and practice, enabling the potential of art in affective-sexual learning to be examined, compared, and theorized across contexts in a sustained and systematic manner. This contributes to moving the field beyond fragmented empirical observations toward deeper theoretical articulation and dialogue.

### 5.2 Implications for research and practice

The generative conditions framework offers an analytic perspective for understanding the role of art in affective–sexual learning and how such learning unfolds generatively within specific contexts.

First, at the level of research orientation, the framework shifts analytic attention from outcome verification to condition-based analysis. Existing studies have often focused on evaluating the effects of arts-based interventions on knowledge, attitudes, or behaviors. The generative conditions framework suggests that such differences are better understood in terms of how relational conditions are configured across contexts.

Second, within this framework, arts-based practice is no longer understood as a replicable sequence of activities, but as a generative space that requires ongoing relational judgment and adjustment. Accordingly, analytic attention shifts away from the design of specific artistic activities toward examining which relational conditions are sustaining the openness and generative potential of the learning space, and which configurations may be leading it to gradually narrow or stagnate. For example, when discussions converge too quickly, expressions become performative, or learners' modes of participation retreat into caution, avoidance, or reticence, these patterns can be understood as signals that generativity is changing. The framework provides a set of diagnostic concepts that enable facilitators to reflect on their role in shaping these conditions and to adjust their modes of intervention, issue framing, or responses in order to sustain generative space.

Finally, the framework supports cross-contextual comparison and interdisciplinary dialogue. Because the generative conditions framework does not depend on specific artistic forms or pedagogical models, but is anchored in a set of transferable relational dimensions, practices across different educational settings can be examined within a shared interpretive structure. This makes it possible to move beyond fragmented accounts shaped by contextual differences and to analyze more precisely how differences among practices arise from variations in condition organization, with resources and activity forms playing a more limited role in explaining these differences. In this way, the framework offers a more robust analytic space for understanding the complexity of adolescents' affective–sexual learning across diverse contexts.

### 5.3 Limitations and directions for future research

It is therefore necessary to clarify the theoretical boundaries, methodological scope, and practical limitations of the generative conditions framework. At the level of conceptual positioning, the framework is intentionally distinguished from art therapy, participatory art practices, and art education models oriented toward methods or interventions. The framework is not directed toward therapeutic aims, symptom reduction, or psychological repair, nor does it assume that arts-based practices necessarily produce clinical or behavioral transformations. Its core concern is not whether art or participatory forms are employed, but how, under specific institutional and relational conditions, such practices form a generative artistic mediating space.

In terms of theoretical function, the generative conditions framework should be understood as an interpretive rather than a normative analytic tool. It does not offer directly replicable teaching models, procedural guidelines, or so-called best practices, nor does it seek to predict or guarantee particular learning outcomes. Instead, its function is to explain how, across different contexts, learners' participation positions, the openness of learning issues, and facilitators' relational roles are configured in ways that support or constrain generative processes. In doing so, the framework provides researchers and practitioners with a conceptual language for identifying and reflecting on how conditions take shape in practice.

From a methodological and temporal perspective, the framework is grounded primarily in theoretical synthesis and a process-oriented interpretive approach, making it particularly suited to qualitative research, comparative case studies, and design-based research. The analysis in this paper focuses on the configuration of relational conditions in the “here and now” of learning processes, and does not systematically address how generative experiences extend, transfer, or accumulate over longer time scales. Future research could employ longitudinal or follow-up designs to further examine how generative conditions evolve over time and what effects these changes may have.

At this point, it is necessary to clarify a potential misinterpretation of the framework's interpretive positioning. While epistemic openness is maintained with regard to meaning-making processes, generativity in this framework does not imply ethical neutrality or the suspension of normative responsibility. In addition, although this paper emphasizes the potential of arts-based mediating spaces to provide psychological safety and symbolic distance, safety should not be understood as emotionally risk-free or entirely comfortable. Especially in affective–sexual learning contexts, arts-based practices may elicit emotional overload or traumatic responses. Such risks do not indicate failure of arts-based practices, but rather highlight the fragility of generative conditions and the responsibility of facilitators to safeguard the space.

At the same time, generative openness may give rise to uneven participation dynamics, where some learners withdraw while others dominate, as well as tensions shaped by cultural, religious, or value-based differences in how affective–sexual issues are understood. These dynamics may introduce moments of discomfort, misunderstanding, or conflict within the learning space. From the perspective of generative conditions, such risks do not in themselves necessitate the closure of openness, but require facilitators to continuously calibrate relational boundaries. This involves attending to signs of distress or exclusion, regulating the pace of interaction, and selectively intervening to maintain psychological safety without prematurely stabilizing meanings or imposing normative resolutions.

Accordingly, the generative conditions framework does not prescribe fixed boundaries for facilitation, but explicitly locates boundary judgment as a form of situated professional responsibility undertaken by facilitators in concrete practice. Given that facilitators' roles in affective–sexual learning are shaped by institutional constraints, ethical requirements, and relational dynamics, such boundaries are difficult to standardize in advance. Future research is therefore needed to explore how professional training, reflective mechanisms, and institutional support can strengthen facilitators' capacity to exercise informed judgment regarding generative conditions and ethical boundaries.

Finally, although this paper does not offer a systematic discussion of curriculum design or professional standards, curricular structures, policy frameworks, and professional training systems constitute important contextual conditions under which generative conditions may be enabled or constrained. Building on the generative conditions framework, future research could further examine how different institutional arrangements shape learners' participation positions, the openness of learning issues, and facilitators' relational roles, as well as compare the possibilities and limitations of sustaining generative artistic mediating spaces across diverse educational contexts and facilitator identities. Future research may also investigate how these relational conditions operate in other domains of arts-based education beyond affective–sexual learning.

## 6 Conclusion

In response to the question of how adolescents engage in affective–sexual learning, this paper proposes and elaborates the theoretical framework of the generative artistic mediating space. The emergence and maintenance of this space depend on the dynamic configuration of three key relational conditions: learners' participation positions, the openness of learning issues, and facilitators' relational roles. From this perspective, the generative conditions framework redirects analytical attention from evaluating the effectiveness of arts-based practices toward examining how relational configurations enable or constrain the ongoing and situated emergence of meaning. It highlights how, even under institutional and relational constraints, generative learning can still be sustained within limited yet meaningful spaces in practice. Accordingly, the contribution of this paper lies in providing an interpretive and condition-oriented analytic lens for understanding how generative learning processes unfold, are sustained, or become constrained across contexts. In doing so, the framework establishes a conceptual point of departure for future research and practice seeking to engage with the relational and contextual complexity of arts-based affective–sexual learning across formal and non-formal environments.
